# Reanalysis of Genomic Sequencing Results in a Clinical Laboratory: Advantages and Limitations

**DOI:** 10.3389/fneur.2020.00612

**Published:** 2020-06-30

**Authors:** Dongju Won, Se Hee Kim, Borahm Kim, Seung-Tae Lee, Hoon-Chul Kang, Jong Rak Choi

**Affiliations:** ^1^Department of Laboratory Medicine, Yonsei University College of Medicine, Seoul, South Korea; ^2^Division of Pediatric Neurology, Department of Pediatrics, Epilepsy Research Institute, Yonsei University College of Medicine, Seoul, South Korea

**Keywords:** reanalysis, next-generation sequencing, epileptic encephalopathy, neurodevelopmental disorder, clinical laboratories

## Abstract

Genetic diagnosis of patients with neurodevelopmental disorders is imperative and a standard clinical practice. Considering the continuous accumulation of data on disease-causing variants, reanalysis of previously established sequencing data is important. Periodic reanalysis of variants with uncertain significance has become mandatory in clinical laboratories. Therefore, to confirm the utility of the reanalysis of targeted gene panel data in clinical laboratories, we re-evaluated the data of two groups of patients who had undergone targeted gene panel testing for neurodevelopmental disorders (*n* = 116) and epileptic encephalopathy (*n* = 384). This reanalysis was based on a reannotation process reflecting updated databases. Six (5.2%) and seven (1.8%) new pathogenic or likely pathogenic variants were identified in these two groups, respectively, attributable to the updated guidelines and *de novo* reports from unrelated patients. Although relatively low, considerable increase in the diagnostic yield was confirmed. We suggest that reanalysis of genetic variants, mainly using changes in databases and updated interpretations, should be implemented as a routine practice in clinical laboratories.

## Introduction

Adoption of massive parallel sequencing has revolutionized the molecular genetic diagnosis of patients with genetically heterogeneous neurodevelopmental disorders. Advancements in technology and development of cost-effective methods have improved the feasibility of multigene panel testing with hundreds of relevant genes and whole-exome sequencing in clinical laboratories. An average diagnostic yield of up to 40% is reported depending on patient group and intensity of analysis (1–5).

Given the continuous accumulation of data on the relationship between gene–disease and variant–disease, periodic reanalysis of the already reported patient results has been considered (6). Several studies have reported that reanalysis using improved bioinformatic tools and updated databases or expanded knowledge on genotype–phenotype correlation is beneficial for the diagnosis of previously unsolved cases (6–14). Furthermore, periodic reanalysis of variants with uncertain significance has now become mandatory in clinical laboratories (15).

The increased diagnostic yield of reanalysis is mostly attributed to newly established gene–disease relationships following initial exome sequencing (16). However, in clinical laboratories that perform the majority of genetic testing using specific gene panels rather than larger whole-exome or whole-genome sequencing, the identification of new pathogenic or likely pathogenic variants based on newly discovered gene–disease relationships is limited. It is also difficult to change bioinformatic tools in clinical laboratories; reanalysis is inevitable, and mainly based on updated databases related to variants or updated guidelines. While next-generation sequencing (NGS) guidelines recommend reanalysis of previously negative NGS data (17), performing reanalysis is difficult in clinical laboratories owing to limited resources. Moreover, the utility of the reanalysis process has been questioned because it is labor-intensive.

Herein, we re-evaluated the established results of targeted gene panel testing in patients with neurodevelopmental disorders, including those with delayed development, intellectual disability, and epileptic encephalopathy, to confirm the applicability of reanalysis of targeted gene panel data in clinical laboratories.

## Materials and Methods

### Data Collection

Gene panel sequencing data were collected from patients who had undergone gene panel testing for neurodevelopmental disorders (*n* = 116) and epileptic encephalopathy (*n* = 384) between January 2017 and July 2018. No pathogenic or likely pathogenic variants were identified. In our clinical laboratory, gene panel testing for epileptic encephalopathy was performed when the patients showed specific epilepsy syndromes, probably related to developmental, and epileptic encephalopathy. Further, gene panel testing for neurodevelopment disorders was performed when the patients presented seizures, not specifically epilepsy syndrome or severe developmental delays. The xGen Inherited Diseases Panel (Integrated DNA Technologies, Coralville, IA, USA) comprising 4,503 genes was used for neurodevelopmental disorders, and a customized gene panel comprising 173 candidate genes (Supplementary Table 1) was used for epileptic encephalopathy. This study was approved by the institutional review board of Severance Hospital, and the requirement for informed consent was waived-off.

### Data Analysis and Interpretation

**Figure 1:**
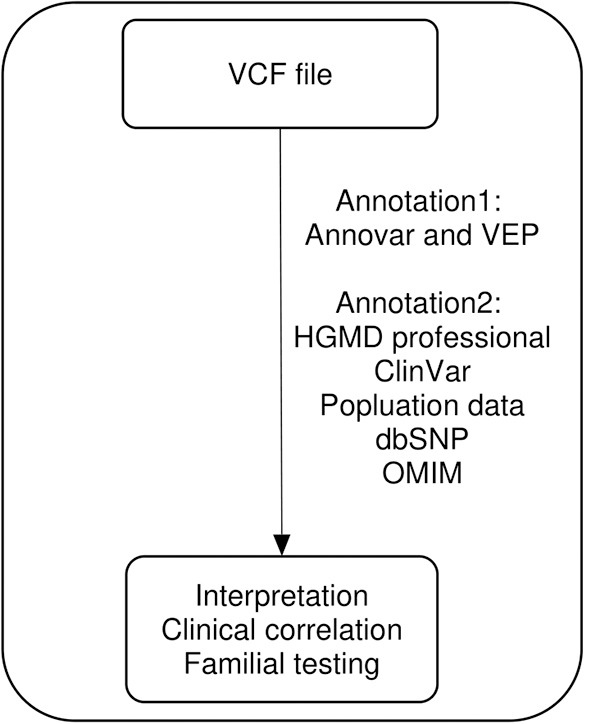
Visual representation of the reanalysis workflow. VCF, variant call format; VEF, Ensembl Variant Effect Predictor; HGMD, Human Gene Mutation Database; OMIM, Online Mendelian Inheritance in Man.

We used a comprehensive custom bioinformatic pipeline that supported a wide range of variants, ranging from single-nucleotide variants to copy-number variants. The flow chart of our bioinformatic pipeline was as previously described (18, 19) without any modification. We only re-annotated the variant call format (VCF) files of patients with no pathogenic or likely pathogenic variants for reanalysis (Figure 1). All annotation processes were automatically performed. The variants from VCF files were first annotated using Annovar and Variant Effect Predictor software to determine their effects on genes, transcripts, protein sequences, and regulatory regions. The variants were then annotated using ClinVar, Online Mendelian Inheritance in Man (OMIM), the Human Gene Mutation Database (HGMD), computational (*in silico*) predictive programs (Mutation Taster, SIFT, PolyPhen-2, PROVEAN), Single Nucleotide Polymorphism Database (dbSNP), 1000 Genome, the Exome Aggregation Consortium, the Genome Aggregation Database, and the Korean Reference Genome Database. These databases were updated four times per year in our laboratory. Following automated annotation, benign or likely benign variants were filtered out by tallying the scores from the frequency of variant-expressing population, *in silico* results, and the literature reported in the databases. We manually checked the automatically re-annotated data and performed parental tests when possible. Reanalysis was performed from May to July 2019. The pathogenicity of variants was classified according to the American College of Medical Genetics and Genomics and the Association for Molecular Pathology (ACMG/AMP) guidelines (17). We used the ClinGen recommendation for *de novo* criteria as indicated in the Sequence Variant Interpretation (SVI) Working Group (https://clinicalgenome.org/working-groups/sequence-variant-interpretation/). This recommendation for *de novo* criteria suggests a point-based system to determine the strength of *de novo* evidence (ACMG/AMP criteria codes PS2 and PM6) based on confirmed vs. assumed status, phenotypic consistency, and number of *de novo* observations. We also used the recommendations by the SVI working group for interpretation of the PVS1 criterion for exon duplication (20).

## Results

### Overall Increase in Diagnostic Yield

In total, 66 of 116 (56.9%) patients who were tested using the neurodevelopmental disorder panel and 231 of 384 (60.2%) patients tested by the gene panel for epilepsy were male. Six pathogenic or likely pathogenic variants were identified in the patients subjected to gene panel testing for neurodevelopmental disorders, accounting for an increase of 5.2% (6/116) in the diagnostic yield. Of the 384 patients previously subjected to gene panel testing for epilepsy, seven new pathogenic or likely pathogenic variants were identified during the course of reanalysis; this accounted for an increase of 1.8% (7/384) in the diagnostic yield. During the initial testing, parental testing had not been conducted for 13 patients owing to the omission or rejection of consent, but it was triggered by changed classification of the variants during reanalysis; we could perform parental testing on three patients (P1, P8, and P13). Except for P11, parents of other 12 patients had shown no symptoms similar to their children. The upgraded variants are described in Table 1. The variants of uncertain significance reported in the initial report for the 13 cases are presented in Supplementary Table 2.

**Table 1:** Characteristics of the newly identified pathogenic or likely pathogenic variants according to the ACMG/AMP guidelines.

**ID**	**Gender**	**Gene panel**	**Gene**	**Inheritance**	**Reference**	**Nucleotide change**	**Amino acid change**	**Zygosity**	**Population frequencies (GnomAD, global)**	ACMG evidence score^*^	**ACMG classification**	**Origin of variant after reanalysis**	**Updated publication after initial report (PMID)**
P1	F	ND	*SCN2A*	AD	NM_001040142.1	c.2932T>C	p.Phe978Leu	Heterozygous	0	PM2, **PM6(1)**, PP2, PP3, PP5	Likely pathogenic	Probable *de novo*	25533962 28191890
P2	M	ND	*ZDHHC9*	X-linked	NM_001008222.2	c.286C>T	p.Arg96Trp	Hemizygous	0	PM2, PP2, PP3, PP5, **PM6_Supporting(0.75)**	Likely pathogenic	Probable *de novo*	28687527 30631761 30402882
P3	F	ND	*ITPR1*	AD	NM_001168272.1	c.800C>T	p.Thr267Met	Heterozygous	0	**PS3**, PM2, PP3, PP5, **PM6_Supporting(0.5)**	Likely pathogenic	Probable *de novo*	29878067 29925855
P4	M	ND	*GLRA1*	AD, AR	NM_001146040.1	c.994G>A	p.Val332Ile	Heterozygous	0.000003983	PM2, PP2, PP3, PP5, **PM6_Supporting(0.5)**	Likely pathogenic	Probable *de novo*	30078784
P5	M	ND	*CACNA1A*	AD	NM_001127221.1	c.1441C>T	p.Arg481Cys	Heterozygous	0.00003186	PM2, **PM6(1)**, PP2, PP3, PP5	Likely pathogenic	Probable *de novo*	28135719 28191890
P6	F	ND	*ALG13*	XLD	NM_001099922.2	c.320A>G	p.Asn107Ser	Heterozygous	0	PM2, **PM6(1.5)**, PP3, PP5,	Likely pathogenic	Probable *de novo*	29314763 30174244 31164858
P7	M	Epilepsy	*CACNA1E*	AD	NM_001205293.1	c.1054G>A	p.Gly352Arg	Heterozygous	0	PM2, PP2, PP3, PP5, **PM6_Supporting(0.5)**	Likely pathogenic	Probable *de novo*	30343943
P8	F	Epilepsy	*ALDH7A1*	AR	NM_001182.4	c.192+3A>T c.1093+5G>T		Heterozygous	0.000004508 0	PM1, PM2, PP3, **PP4**, **PP5** PM2, PP3, **PP4**	Likely pathogenic Uncertain significance	Probably from each parent	ClinVar in 2017
P9	M	Epilepsy	*GNAO1*	AD	NM_020988.2	c.118G>T	p.Gly40Trp	Heterozygous	0	PM2, PP3, PP5, **PM6(1)**	Likely pathogenic	Probable *de novo*	29390993 30682224
P10	M	Epilepsy	*SCN8A*	AD	NM_014191.3	c.1099A>G	p.Met367Val	Heterozygous	0	PM2, PP2, PP3, PP5, **PM6_Supporting(0.5)**	Likely pathogenic	Probable *de novo*	29655203
P11	M	Epilepsy	*GRIN2A*	AD	NM_000833.3	exon 3,4 duplication		Heterozygous	Not Applicable	**PVS1_Strong**, PM2, PP4, PP5	Likely pathogenic	Probable maternal inheritance	30544257
P12	F	Epilepsy	*SCN8A*	AD	NM_014191.3	c.2549G>A	p.Arg850Gln	Heterozygous	0	PM2, PP2, PP3, PP5, **PM6(1)**	Likely pathogenic	Probable *de novo*	29186148 29720203
P13	M	Epilepsy	*KCNC1*	AD	NM_001112741.1	c.1262C>T	p.Ala421Val	Heterozygous	0	**PS3**, PM2, PP3, PP5, **PM6(1)**	Pathogenic	Probable *de novo*	31353862 31353855

*ND, neurodevelopment; AD, autosomal dominant; AR, autosomal recessive; XLD, X-linked dominant; PVS, pathogenic very strong; PS, pathogenic strong; PM, pathogenic moderate; PP, pathogenic supporting*.

* *Codes written in bold imply new evidence after reanalysis*.

*The numbers in parentheses mean total points awarded according to ClinGen Sequence Variant Interpretation Recommendation for de novo Criteria. The phenotypic consistency in P1 and P4-P13 with epilepsy was considered “phenotype consistent with gene but not highly specific” and phenotypic consistency in P2 and P3 without epilepsy was considered “Phenotype consistent with gene but not highly specific and high genetic heterogeneity”*.

### Updated Guidelines, New Variant–Disease Associations, and Phenotyping

The SVI Working Group suggested in 2018 that the discovery of a *de novo* variant in unrelated patients might allow its prediction in patients with unaffected parents. The variants from P1-P7, P9-P10, and P12-P13 with unaffected parents were *de novo* in unrelated patients in the new literature. Therefore, they were given various levels of pathogenic evidence by the sum of points (Table 1). We contacted their parents to explain the results of reanalysis and received consent for parental testing from the parents of P1 and P13. The variants from P1 and P13 were confirmed to be *de novo*. In addition, the variants from P3 and P13 were associated with the PS3 code, owing to the new literature on decreased protein function. The P3 variant in *ITPR1*, a gene encoding a calcium channel that modulates intracellular calcium signaling, was shown to decrease calcium ion release in the endoplasmic reticulum (21). The P13 variant in *KCNC1*, encoding a highly conserved subunit of a potassium ion channel, was demonstrated to decrease the amplitude of current (22). In the case of *ALDH7A1* (P8), which is associated with the recessive disease pyridoxine-dependent epilepsy, two variants were identified near the intronic junction; these were not canonical splice variants but were reported as variants of uncertain significance owing to the lack of evidence. The variant c.192+3A>T (NM_001182.4) was thought to be likely pathogenic in ClinVar in October 2017 after our initial report and was assigned the PP5 code. After additional phenotyping that identified decreased seizures with pyridoxine administration, the variant was assigned the PP4 code and deemed to be likely pathogenic. Considering the reinterpretation of c.192+3A>T variant to be likely pathogenic, the variant c.1093+5G>T was likely to receive PM3 score owing to its *trans* position with the variant c.192+3A>T. The patient's parents were informed of the reanalysis results and the need for a parent test. The variant c.1093+5G>T in *ALDH7A1* was actually identified in *trans* with c.192+3A>T following parental testing. Exon duplication, unlike exon deletion, has not been described in detail in the ACMG/AMP guidelines published in 2015; thus, exon 3,4 duplication in *GRIN2A* in P11 was reported as a variant of uncertain significance (Supplementary Figure 1). However, in 2018, new guidelines for PVS1 were released (20), and the code PVS1_Strong was assigned for the exon duplication in *GRIN2A* because the reading frame was thought to be disrupted and the occurrence of non-sense-mediated decay was predicted.

### Changed Genetic Inheritance or Updated Gene–Disease Associations

As P6 was a female patient, one missense variant in *ALG13*, known to be inherited in an X-linked recessive mode, was underestimated in our initial report. However, the mode of inheritance in *ALG13* changed from X-linked recessive to X-linked dominant. Therefore, the likelihood of disease association of the variant c.320A>G (NM_001099922.2) in *ALG13* increased in P6. It was deemed to be likely pathogenic, as it was reported as *de novo* in other patients. The initial diagnostic test was performed, and *CACNA1E* encoding a subunit of a calcium channel was considered a candidate for epileptic encephalopathy; however, the OMIM database did not clearly report this gene and the related disease. Therefore, we conservatively interpreted the *CACNA1E* missense variant as a variant of uncertain significance (P7). *CACNA1E* was linked to epileptic encephalopathy in OMIM in 2019 (CACNA1E, OMIM# 601013); thus, the *CACNA1E* variant c.1054G>A (NM_001205293.1) in P7 became more noticeable and was interpreted as a likely pathogenic *de novo* variant from unrelated patients.

## Discussion

Neurodevelopmental disorders, including epileptic encephalopathy, affect more than 3% children worldwide (23). The genetic diagnosis of these diseases is gaining importance, and NGS technology with massive parallel sequencing has become the standard clinical practice. Approximately 250 novel gene–disease and 9,200 novel variant–disease associations are reported every year (6). Therefore, the significance of reanalysis of negative results obtained from previous rounds of NGS cannot be overemphasized.

Several reports have described the reanalysis of whole-exome or clinical-exome sequencing data, showing varying yields from approximately 10% to 30% performed by applying improved bioinformatic pipelines or searching for newly discovered disease-associated genes (6–14). To achieve maximum diagnostic yield, realignment with upgraded tools through the inspection and clarification of patients' symptoms and signs, data sharing and collaboration with other institutes, and reannotation of variants using updated databases should be periodically performed. However, the process of reanalysis in clinical laboratories is limited because it is time consuming and labor-intensive. Furthermore, frequent changes in analytical pipelines may hinder routine work, and patients can only be contacted when they visit centers for appointment. In addition, detailed phenotyping is often difficult, given the limited treatment time available.

In the present study, we performed reanalysis of the previous data using updated guidelines, new variant–disease associations with some phenotyping, new genetic inheritance, and updated gene–disease associations, all of which contributed to the increase in the diagnostic yield. We reanalyzed the results using two gene panels comprising 173 and 4,503 genes based on limited conditions. For efficient bioinformatic analysis, we incepted with VCF files and used automated annotation programs (Figure 1). This process allowed us to reduce labor and time because it excluded the need to manually search the databases. We identified six new pathogenic or likely pathogenic variants (5.2%) in 116 patients who underwent neurodevelopmental disorder panel testing and seven new pathogenic or likely pathogenic variants (1.8%) in 384 patients who underwent epileptic encephalopathy panel testing. The increase in the diagnostic yield was relatively low, possibly due to the lack of updates with respect to newly established gene–disease associations. It may also be attributed to the fact that we performed panel sequencing, not exome sequencing, without changing the previously used bioinformatic tools and without in-depth phenotyping. Although the cost reduction for large-scale genome sequencing may increase the utilization of whole-exome sequencing as a routine practice, many clinical laboratories still employ gene panels with a limited number of target genes (24). The identification of 13 new pathogenic or likely pathogenic variants using targeted gene panel results demonstrates the benefit of this approach, considering the limited time and resources available in clinical laboratories.

We learnt several lessons from the reanalysis of targeted panel sequencing data at the clinical laboratory level. First, it is important to keep a track of the relevant guidelines for the correct interpretation of variants. The ACMG/AMP guidelines published in 2015 provide information on variant interpretation necessary during the initial testing but may lack some explanation. The updated recommendation for *de novo* criteria highlighted that *de novo* reports from unrelated patients could help interpret the variants. In addition, the updated guidelines for the PVS1 code provided us with the evidence for the interpretation of exon duplication.

Second, even during reanalysis, identifying variants related to the cause of an underlying disease may be useful to treat patients. In the case of P8, *ALDH7A1* variants associated with pyridoxine-dependent epilepsy were thought to be related to patient's seizures, and the administration of pyridoxine improved seizures in this patient. In the case of P4, clonazepam that was found to be effective in patients with the same *GLRA1* variant related to hyperekplexia (25) could be administered to our patient.

Third, it is advantageous to include as many relevant genes as possible during the designing of gene panel. During reanalysis, the yield of our gene panel for neurodevelopment disorders with a higher number of genes was higher than that of the gene panel for epileptic encephalopathy. *ZDHHC9, ITPR1*, and *GLRA1* showed meaningful variants in the gene panel for neurodevelopment disorders during reanalysis but were not actually included in the gene panel for epileptic encephalopathy.

Our study has a few limitations. The reanalysis was performed on two different set of panels, not by exome sequencing, owing to the nature of the clinical laboratory. Hence, it was difficult to reveal the variants upgraded from the discovery of new gene–disease associations. In addition, only unsolved cases were subject to reanalysis. Future studies should be conducted on previously pathogenic or likely pathogenic variants.

In conclusion, we reanalyzed the data obtained using a small and large gene panel, starting with VCF files. The main factors were updated guidelines and *de novo* reports from other patients. Although relatively low, the increase in the diagnostic yield was considerable. This approach may encourage the implementation of data reanalysis as a routine process in clinical laboratories.

## Data Availability Statement

The datasets generated for this study can be found in the Supplementary Material.

## Ethics Statement

The studies involving human participants were reviewed and approved by Severance Hospital, Institutional Review Board. Written informed consent from the participants' legal guardian/next of kin was not required to participate in this study in accordance with the national legislation and the institutional requirements. Written informed consent was not obtained from the minor(s)' legal guardian/next of kin for the publication of any potentially identifiable images or data included in this article.

## Author Contributions

DW and SK analyzed the data and wrote the paper. BK and S-TL verified the analytical method and aided in interpreting the results. JC and H-CK designed the study and supervised the findings of the work. All authors discussed the results and contributed to the final manuscript.

## Conflict of Interest

The authors declare that the research was conducted in the absence of any commercial or financial relationships that could be construed as a potential conflict of interest.

## References

[1] Retterer K Juusola J Cho MT Vitazka P Millan F Gibellini F. Clinical application of whole-exome sequencing across clinical indications. Genet Med. (2016) 18:696–704. 10.1038/gim.2015.148 26633542

[2] Zhu X Petrovski S Xie P Ruzzo EK Lu YF McSweeney KM. Whole-exome sequencing in undiagnosed genetic diseases: interpreting 119 trios. Genet Med. (2015) 17:774–81. 10.1038/gim.2014.191 25590979 PMC4791490

[3] Lee H Deignan JL Dorrani N Strom SP Kantarci S Quintero-Rivera F. Clinical exome sequencing for genetic identification of rare Mendelian disorders. Jama. (2014) 312:1880–7. 10.1001/jama.2014.14604 25326637 PMC4278636

[4] Farwell KD Shahmirzadi L El-Khechen D Powis Z Chao EC Tippin Davis B. Enhanced utility of family-centered diagnostic exome sequencing with inheritance model-based analysis: results from 500 unselected families with undiagnosed genetic conditions. Genet Med. (2015) 17:578–86. 10.1038/gim.2014.154 25356970

[5] Yang Y Muzny DM Reid JG Bainbridge MN Willis A Ward PA. Clinical whole-exome sequencing for the diagnosis of mendelian disorders. N Engl J Med. (2013) 369:1502–11. 10.1056/NEJMoa1306555 24088041 PMC4211433

[6] Wenger AM Guturu H Bernstein JA Bejerano G Systematic reanalysis of clinical exome data yields additional diagnoses: implications for providers. Genet Med. (2017) 19:209–14. 10.1038/gim.2016.88 27441994

[7] Jalkh N Corbani S Haidar Z Hamdan N Farah E Abou Ghoch J. The added value of WES reanalysis in the field of genetic diagnosis: lessons learned from 200 exomes in the Lebanese population. BMC Med Genomics. (2019) 12:11. 10.1186/s12920-019-0474-y 30665423 PMC6341681

[8] Al-Nabhani M Al-Rashdi S Al-Murshedi F Al-Kindi A Al-Thihli K Al-Saegh A. Reanalysis of exome sequencing data of intellectual disability samples: yields and benefits. Clin Genet. (2018) 94:495–501. 10.1111/cge.13438 30125339

[9] Wright CF McRae JF Clayton S Gallone G Aitken S FitzGerald TW. Making new genetic diagnoses with old data: iterative reanalysis and reporting from genome-wide data in 1,133 families with developmental disorders. Genet Med. (2018) 20:1216–23. 10.1038/gim.2017.246 29323667 PMC5912505

[10] Alfares A Aloraini T Subaie LA Alissa A Qudsi AA Alahmad A. Whole-genome sequencing offers additional but limited clinical utility compared with reanalysis of whole-exome sequencing. Genet Med. (2018) 20:1328–33. 10.1038/gim.2018.41 29565419

[11] Nambot S Thevenon J Kuentz P Duffourd Y Tisserant E Bruel AL. Clinical whole-exome sequencing for the diagnosis of rare disorders with congenital anomalies and/or intellectual disability: substantial interest of prospective annual reanalysis. Genet Med. (2018) 20:645–54. 10.1038/gim.2017.162 29095811

[12] Baker SW Murrell JR Nesbitt AI Pechter KB Balciuniene J Zhao X. Automated clinical exome reanalysis reveals novel diagnoses. J Mol Diagnost. (2019) 21:38–48. 10.1016/j.jmoldx.2018.07.008 30577886

[13] Ewans LJ Schofield D Shrestha R Zhu Y Gayevskiy V Ying K. Whole-exome sequencing reanalysis at 12 months boosts diagnosis and is cost-effective when applied early in Mendelian disorders. Genet Med. (2018) 20:1564–74. 10.1038/gim.2018.39 29595814

[14] Li J Gao K Yan H Xiangwei W Liu N Wang T. Reanalysis of whole exome sequencing data in patients with epilepsy and intellectual disability/mental retardation. Gene. (2019) 700:168–75. 10.1016/j.gene.2019.03.037 30904718

[15] Deignan JL Chung WK Kearney HM Monaghan KG Rehder CW Chao EC Points to consider in the reevaluation and reanalysis of genomic test results: a statement of the American College of Medical Genetics and Genomics (ACMG). Genet Med. (2019) 21:1267–70. 10.1038/s41436-019-0478-1 31015575 PMC6559819

[16] Liu P Meng L Normand EA Xia F Song X Ghazi A. Reanalysis of Clinical Exome Sequencing Data. N Engl J Med. (2019) 380:2478–80. 10.1056/NEJMc1812033 31216405 PMC6934160

[17] Richards S Aziz N Bale S Bick D Das S Gastier-Foster J. Standards and guidelines for the interpretation of sequence variants: a joint consensus recommendation of the American College of Medical Genetics and Genomics and the Association for Molecular Pathology. Genet Med. (2015) 17:405–24. 10.1038/gim.2015.30 25741868 PMC4544753

[18] Kim SH Kim B Lee JS Kim HD Choi JR Lee ST. Proband-only clinical exome sequencing for neurodevelopmental disabilities. Pediatr Neurol. (2019) 99:47–54. 10.1016/j.pediatrneurol.2019.02.017 30952489

[19] Rim JH Kim SH Hwang IS Kwon SS Kim J Kim HW. Efficient strategy for the molecular diagnosis of intractable early-onset epilepsy using targeted gene sequencing. BMC Med Genomics. (2018) 11:6. 10.1186/s12920-018-0320-7 29390993 PMC5796507

[20] Abou Tayoun AN Pesaran T DiStefano MT Oza A Rehm HL Biesecker LG. Recommendations for interpreting the loss of function PVS1 ACMG/AMP variant criterion. Hum Mutat. (2018) 39:1517–24. 10.1002/humu.23626 30192042 PMC6185798

[21] Synofzik M Helbig KL Harmuth F Deconinck T Tanpaiboon P Sun B. De novo ITPR1 variants are a recurrent cause of early-onset ataxia, acting via loss of channel function. Eur J Hum Genet. (2018) 26:1623–34. 10.1038/s41431-018-0206-3 29925855 PMC6189112

[22] Cameron JM Maljevic S Nair U Aung YH Cogne B Bezieau S. Encephalopathies with KCNC1 variants: genotype-phenotype-functional correlations. Ann Clin Transl Neurol. (2019) 6:1263–72. 10.1002/acn3.50822 31353855 PMC6649578

[23] Tarlungeanu DC Novarino G Genomics in neurodevelopmental disorders: an avenue to personalized medicine. Exp Mol Med. (2018) 50:100. 10.1038/s12276-018-0129-7 30089840 PMC6082867

[24] Santani A Murrell J Funke B Yu Z Hegde M Mao R. Development and validation of targeted next-generation sequencing panels for detection of germline variants in inherited diseases. Arch Pathol Lab Med. (2017) 141:787–97. 10.5858/arpa.2016-0517-RA 28322587

[25] Wadi L Medlej Y Obeid M A child with hyperekplexia and epileptic myoclonus. Epileptic Disord. (2018) 20:279–82. 10.1684/epd.2018.0986 30078784

