# Supplementary material for: Reanalysis of Genomic Sequencing Results in a Clinical Laboratory: Advantages and Limitations
Source: Front Neurol. 2020 Jun 30;11:612. doi: 10.3389/fneur.2020.00612 (PMC7338758; doi:10.3389/fneur.2020.00612)

Supplementary Material

**Supplementary Table 1.** Genes included in the targeted gene panel for epilepsy.

*ADSL, FOXG1, LIAS, MAGI2, MBD5, MFSD8, NHLRC1, OPHN1, PIGQ, PPT1, PRICKLE1, GABBR2, PRICKLE2, PRRT2, PURA, SCARB2, SCN1B, SCN3A, SLC9A6, SMARCA2, SRPX2, ST3GAL5, GABRA1, SYN1, TBL1XR1, TCF4, TNK2, TPP1, TSEN54, UBE2A, UBE3A, WDR62, ZEB2, GABRB3, ASNS, BRAT1, COL4A1, GABRG2, GNAO1, GRIN1, GRIN2A, GRIN2B, HCN1, ALG13, HNRNPU, IQSEC2, KANSL1, KCNA2, KCNB1, KCNH5, KCNQ2, KCNQ3, KCNT1, KPNA7, ARX, MECP2, MEF2C, NRXN1, PCDH19, PIGA, PLCB1, PNKP, POLG, QARS, SCN1A, CACNA1A, SCN2A, SCN8A, SCN9A, SETBP1, SLC6A1, SLC13A5, SLC25A22, SPTAN1, ST3GAL3, STX1B, CASK, STXBP1, SYNGAP1, SZT2, TBC1D24, WWOX, GLDC, DOCK7, CDKL5, NECAP1, AARS, SIK1, ARHGEF9, ABAT, ACY1, ADGRV1, ARHGEF15, ASPM, ATP13A2, CHD2, ATP6AP2, CACNB4, CASR, CHRNA2, CHRNA4, CHRNA7, CHRNB2, CLCN4, CLN3, CLN5, DNM1, CLN6, CLN8, CNTNAP2, CSTB, CTSD, CTSF, DNAJC5, DYRK1A, EPM2A, FARS2, EEF1A2, GOSR2, GRN, HCN4, KCNA1, KCNC1, KCNJ10, KCNJ11, KCNMA1, KCTD7, LGI1, FOLR1, SLC2A1, GAMT, GATM, SLC6A8, BTD, HLCS, SLC19A3, PHGDH, SLC22A5, SLC46A1, CPT1A, CPT1B, CPT2, SLC25A20, SLC25A29, ACADM, ACADL, ACADS, HADHA, HADH, ALPL, PC, ALAD, ALAS2, CPOX, FECH, HFE, HMBS, PPOX, UROD, UROS, ALDH4A1, MTHFR, MTR, MTRR, CBS, MMADHC, PAH, PRODH, PNPO, ALDH7A1, AMT, GCSH, CACNA1E*

**Supplementary Table 2.** List of variants of uncertain significance first reported in 13 cases upgraded after reanalysis. The ones in bold were updated as pathogenic or likely pathogenic after reanalysis.

| ID | Gene panel | Class | Gene | Reference | Nucleotide change | Amino acid change | Zygosity |
| --- | --- | --- | --- | --- | --- | --- | --- |
| P1 | Neuro development | **VOUS** | ***SCN2A*** | **NM_001040142.1** | **c.2932T>C** | **p.Phe978Leu** | **Heterozygous** |
|  |  | VOUS | *F11* | NM_000128.3 | c.1556G>A | p.Trp519Ter | Heterozygous |
|  |  | VOUS | *DHCR7* | NM_001360.2 | c.907G>A | p.Gly303Arg | Heterozygous |
|  |  | VOUS | *CFTR* | NM_000492.3 | c.2735C>T | p.Ser912Leu | Heterozygous |
|  |  | VOUS | *DUOX2* | NM_014080.4 | c.3478_3480del | p.Leu1160del | Heterozygous |
|  |  | VOUS | *INSR* | NM_000208.2 | c.2572A>G | p.Thr858Ala | Heterozygous |
|  |  | VOUS | *FZD4* | NM_012193.3 | c.205C>T | p.His69Tyr | Heterozygous |
|  |  | VOUS | *AKT1* | NM_001014432.1 | c.1373T>C | p.Met458Thr | Heterozygous |
|  |  | VOUS | *PON1* | NM_000446.5 | c.381G>T | p.Met127Ile | Heterozygous |
|  |  | VOUS | *CNTNAP2* | NM_014141.5 | c.1385G>A | p.Arg462His | Heterozygous |
|  |  | VOUS | *TLR1* | NM_003263.3 | c.1279delA | p.Ser427ValfsTer2 | Heterozygous |
|  |  | VOUS | *ATN1* | NM_001007026.1 | c.1462_1476dup | p.Gln498_Gln502dup | Heterozygous |
|  |  | VOUS | *ALMS1* | NM_015120.4 | c.36_41delGGAGGA insGGAGGAGGA | p.Glu28dup | Heterozygous |
|  |  | VOUS | *TTN* | NM_133379.4 | c.15267_15332del | p.Thr5091_Glu5112del | Heterozygous |
|  |  | VOUS | *PIGN* | NM_176787.4 | c.963G>A | p.= | Heterozygous |
|  |  | VOUS | *CDK5RAP2* | NM_018249.5 | c.4425G>A | p.= | Heterozygous |
|  |  | VOUS | *FLNA* | NM_001110556.1 | c.4533T>C | p.= | Heterozygous |
| P2 | Neuro development | **VOUS** | ***ZDHHC9*** | **NM_001008222.2** | **c.286C>T** | **p.Arg96Trp** | **Hemizygous** |
|  |  | VOUS | *NSD1* | NM_022455.4 | c.2608A>G | p.Arg870Gly | Heterozygous |
|  |  | VOUS | *BEST1* | NM_004183.3 | c.584C>T | p.Ala195Val | Heterozygous |
|  |  | VOUS | *CLCN1* | NM_000083.2 | c.313C>T | p.Arg105Cys | Heterozygous |
|  |  | VOUS | *SCN10A* | NM_006514.2 | c.4205T>C | p.Ile1402Thr | Heterozygous |
|  |  | VOUS | *PDE6C* | NM_006204.3 | c.1590delT | p.Phe530LeufsTer3 | Heterozygous |
| P3 | Neuro development | **VOUS** | ***ITPR1*** | **NM_001168272.1** | **c.800C>T** | **p.Thr267Met** | **Heterozygous** |
|  |  | VOUS | *TRPM2* | NM_003307.3 | c.1461delG | p.Met488Ter | Heterozygous |
|  |  | VOUS | *GSN* | NM_000177.4 | c.11_38del | p.His4ArgfsTer86 | Heterozygous |
|  |  | VOUS | *MLC1* | NM_015166.3 | c.35A>C | p.Tyr12Ser | Heterozygous |
| P4 | Neuro development | **VOUS** | ***GLRA1*** | **NM_001146040.1** | **c.994G>A** | **p.Val332Ile** | **Heterozygous** |
|  |  | VOUS | *MED13L* | NM_015335.4 | c.4949A>C | p.Gln1650Pro | Heterozygous |
|  |  | VOUS | *CCBE1* | NM_133459 | exon 3-11 deletion |  | Heterozygous |
|  |  | VOUS | *ABCC8* | NM_000352.3 | c.307C>T | p.His103Tyr | Heterozygous |
|  |  | VOUS | *GLRA1* | NM_001146040.1 | c.994G>A | p.Val332Ile | Heterozygous |
|  |  | VOUS | *ATP7A* | NM_000052.5 | c.15G>A | p.Met5Ile | Hemizygous |
|  |  | VOUS | *DYSF* | NM_003494.3 | c.663+1G>C |  | Heterozygous |
|  |  | VOUS | *PLA2G6* | NM_003560.2 | c.266C>A | p.Ser89Tyr | Heterozygous |
|  |  | VOUS | *MKKS* | NM_170784.2 | c.757T>C | p.Ser253Pro | Heterozygous |
| P5 | Neuro development | **VOUS** | ***CACNA1A*** | **NM_001127221.1** | **c.1441C>T** | **p.Arg481Cys** | **Heterozygous** |
|  |  | VOUS | *LINS1* | NM_001040616.2 | c.982dupC | p.His328ProfsTer12 | Heterozygous |
|  |  | VOUS | *GUCY2D* | NM_000180.3 | c.2649delT | p.Phe883LeufsTer13 | Heterozygous |
|  |  | VOUS | *RYR2* | NM_001035.2 | c.1561G>A | p.Glu521Lys | Heterozygous |
|  |  | VOUS | *KIF21A* | NM_001173464.1 | c.3115T>C | p.Tyr1039His | Heterozygous |
|  |  | VOUS | *LRP5* | NM_002335.2 | c.1996G>A | p.Asp666Asn | Heterozygous |
|  |  | VOUS | *COL4A2* | NM_001846.2 | c.5033C>T | p.Thr1678Ile | Heterozygous |
|  |  | VOUS | *MAF* | NM_005360.4 | c.454G>A | p.Gly152Ser | Heterozygous |
|  |  | VOUS | *NAGLU* | NM_000263.3 | c.1322C>T | p.Thr441Met | Heterozygous |
|  |  | VOUS | *AVPR2* | NM_000054.4 | c.749G>A | p.Gly250Glu | Hemizygous |
|  |  | VOUS | *AVPR2* | NM_000054.4 | c.751C>G | p.Arg251Gly | Hemizygous |
|  |  | VOUS | *ALDOA* | NM_000034.3 | c.661C>A | p.His221Asn | Heterozygous |
| P6 | Neuro development | VOUS | *KCNJ12* | NM_021012.4 | c.438C>A | p.Tyr146Ter | Heterozygous |
|  |  | VOUS | *UGT1A4* | NM_007120.2 | c.867+1G>T |  | Heterozygous |
|  |  | **VOUS** | ***ALG13*** | **NM_001099922.2** | **c.320A>G** | **p.Asn107Ser** | **Heterozygous** |
|  |  | VOUS | *EWSR1* | NM_001163287.1 | c.1030delG | p.Val344TyrfsTer40 | Heterozygous |
|  |  | VOUS | *ABCC8* | NM_000352.3 | c.4035G>T | p.Gln1345His | Heterozygous |
|  |  | VOUS | *SETX* | NM_015046.5 | c.3815A>G | p.Lys1272Arg | Heterozygous |
|  |  | VOUS | *ARID1A* | NM_006015.4 | c.4906C>T | p.Arg1636Trp | Heterozygous |
|  |  | VOUS | *CHD8* | NM_001170629.1 | c.676G>A | p.Ala226Thr | Heterozygous |
|  |  | VOUS | *MEFV* | NM_000243.2 | c.390C>G | p.Asn130Lys | Heterozygous |
|  |  | VOUS | *ADK* | NM_006721.3 | c.2T>A | p.Met1? | Heterozygous |
|  |  | VOUS | *SUCLG1* | NM_003849.3 | c.962C>T | p.Ala321Val | Heterozygous |
|  |  | VOUS | *NT5C3A* | NM_001002010.2 | c.11C>T | p.Pro4Leu | Heterozygous |
|  |  | VOUS | *ACADSB* | NM_001609.3 | c.655G>A | p.Val219Met | Heterozygous |
|  |  | VOUS | *RECQL4* | NM_004260.3 | c.3457G>A | p.Gly1153Arg | Heterozygous |
| P7 | Epilepsy | **VOUS** | ***CACNA1E*** | **NM_001205293.1** | **c.1054G>A** | **p.Gly352Arg** | **Heterozygous** |
|  |  | VOUS | *SCN1A* | NM_006920.4 | c.1193C>T | p.Thr398Met | Heterozygous |
|  |  | VOUS | *MAGI2* | NM_012301.3 | c.1409-8delT |  | Heterozygous |
|  |  | VOUS | *DOCK7* | NM_033407.3 | c.184G>A | p.Asp62Asn | Heterozygous |
|  |  | VOUS | *ADGRV1* | NM_032119.3 | c.4171G>A | p.Glu1391Lys | Heterozygous |
|  |  | VOUS | *GABRG2* | NM_198903.2 | c.24C>A | p.Ser8Arg | Heterozygous |
| P8 | Epilepsy | **VOUS** | ***ALDH7A1*** | **NM_001182.4** | **c.192+3A>T** |  | **Heterozygous** |
|  |  | **VOUS** | ***ALDH7A1*** | **NM_001182.4** | **c.1093+5G>T** |  | **Heterozygous** |
|  |  | VOUS | *ADGRV1* | NM_032119.3 | c.929G>A | p.Gly310Glu | Heterozygous |
|  |  | VOUS | *ACADM* | NM_000016.4 | c.*5dup |  | Heterozygous |
|  |  | VOUS | *HMBS* | NM_000190.3 | c.772-17A>G |  | Heterozygous |
|  |  | VOUS | *KCNJ10* | NM_002241.4 | c.937C>T | p.Pro313Ser | Heterozygous |
|  |  | VOUS | *GCSH* | NM_004483.4 | c.-1C>T |  | Heterozygous |
|  |  | VOUS | *CACNA1E* | NM_001205293.1 | c.969A>G | p.Gly323= | Heterozygous |
|  |  | VOUS | *GRIN2B* | NM_000834.3 | c.609C>T | p.Leu203= | Heterozygous |
|  |  | VOUS | *KCNH5* | NM_139318.4 | c.2616C>T | p.Asp872= | Heterozygous |
|  |  | VOUS | *CHD2* | NM_001271.3 | c.2706T>C | p.His902= | Heterozygous |
|  |  | VOUS | *GRIN2C* | NM_000835.4 | c.2451C>T | p.Gly817= | Heterozygous |
|  |  | VOUS | *SOWAHA* | NM_175873.5 | c.1554G>A | p.Lys518= | Heterozygous |
| P9 | Epilepsy | **VOUS** | ***GNAO1*** | **NM_020988.2** | **c.118G>T** | **p.Gly40Trp** | **Heterozygous** |
|  |  | VOUS | *CHD2* | NM_001271.3 | c.3298G>C | p.Ala1100Pro | Heterozygous |
|  |  | VOUS | *GRIN2A* | NM_000833.4 | c.1006C>A | p.Pro336Thr | Heterozygous |
|  |  | VOUS | *HFE* | NM_000410.3 | c.*296A>T |  | Heterozygous |
|  |  | VOUS | *SLC19A3* | NM_025243.3 | c.982G>T | p.Ala328Ser | Heterozygous |
|  |  | VOUS | *DLX6* | NM_005222.3 | c.93_98del | p.Gln43_Gln44del | Heterozygous |
|  |  | VOUS | *HCN3* | NM_020897.2 | c.1083G>A | p.Gln361= | Heterozygous |
|  |  | VOUS | *NRXN3* | NM_004796.5 | c.676T>C | p.Trp226Arg | Heterozygous |
| P10 | Epilepsy | **VOUS** | ***SCN8A*** | **NM_014191.3** | **c.1099A>G** | **p.Met367Val** | **Heterozygous** |
|  |  | VOUS | *PC* | NM_001040716.1 | c.3435G>T | p.Met1145Ile | Heterozygous |
|  |  | VOUS | *GLDC* | NM_000170.2 | c.668C>G | p.Pro223Arg | Heterozygous |
|  |  | VOUS | *SLC19A3* | NM_025243.3 | c.1132A>G | p.Ile378Val | Heterozygous |
|  |  | VOUS | *CHRNA4* | NM_000744.6 | c.414G>A | p.= | Heterozygous |
|  |  | VOUS | *SZT2* | NM_015284.3 | c.7392G>A | p.= | Heterozygous |
| P11 | Epilepsy | **VOUS** | ***GRIN2A*** | **NM_000833.3** | **exon 3,4 duplication** |  | **Heterozygous** |
|  |  | VOUS | *MBD5* | NM_018328.4 | c.4450C>T | p.Pro1484Ser | Heterozygous |
|  |  | VOUS | *WDR62* | NM_001083961.1 | c.3083-6T>C |  | Heterozygous |
|  |  | VOUS | *WDR62* | NM_001083961.1 | c.4021G>A | p.Gly1341Ser | Heterozygous |
|  |  | VOUS | *KCNJ10* | NM_002241.4 | c.142C>T | p.Arg48Cys | Heterozygous |
|  |  | VOUS | *COL4A1* | NM_001845.4 | c.1470C>T | p.Phe490= | Heterozygous |
|  |  | VOUS | *KCNQ2* | NM_172107.2 | c.855C>G | p.Pro285= | Heterozygous |
|  |  | VOUS | *ALDH4A1* | NM_170726.2 | c.199G>A | p.Val67Met | Heterozygous |
|  |  | VOUS | *CPT1B* | NM_001145135.1 | c.1584G>A | p.Ala528= | Heterozygous |
|  |  | VOUS | *NRXN1* | NM_001135659.1 | c.4180A>T | p.Thr1394Ser | Heterozygous |
|  |  | VOUS | *PIGQ* | NM_004204.3 | c.1437C>T | p.Ala479= | Heterozygous |
|  |  | VOUS | *TNK2* | NM_005781.4 | c.1764C>G | p.Ser588= | Heterozygous |
|  |  | VOUS | *TNK2* | NM_005781.4 | c.2243G>A | p.Arg748Gln | Heterozygous |
|  |  | VOUS | *ATP13A2* | NM_022089.2 | c.106-5C>T |  | Heterozygous |
| P12 | Epilepsy | **VOUS** | ***SCN8A*** | **NM_014191.3** | **c.2549G>A** | **p.Arg850Gln** | **Heterozygous** |
|  |  | VOUS | *PNKP* | NM_007254.3 | c.56C>T | p.Ala19Val | Heterozygous |
|  |  | VOUS | *SLC25A22* | NM_001191061.1 | c.194T>C | p.Met65Thr | Heterozygous |
|  |  | VOUS | *ALDH4A1* | NM_170726.2 | c.643A>G | p.Ile215Val | Heterozygous |
|  |  | VOUS | *ATP13A2* | NM_022089.2 | c.106-5C>T |  | Heterozygous |
| P13 | Epilepsy | **VOUS** | ***KCNC1*** | **NM_001112741.1** | **c.1262C>T** | **p.Ala421Val** | **Heterozygous** |
|  |  | VOUS | *KANSL1* | NM_001193466.1 | exon 3_4 duplication |  | Heterozygous |

**Supplementary Figure 1.** Exon 3-4 duplication of *GRIN2A* found in P11.


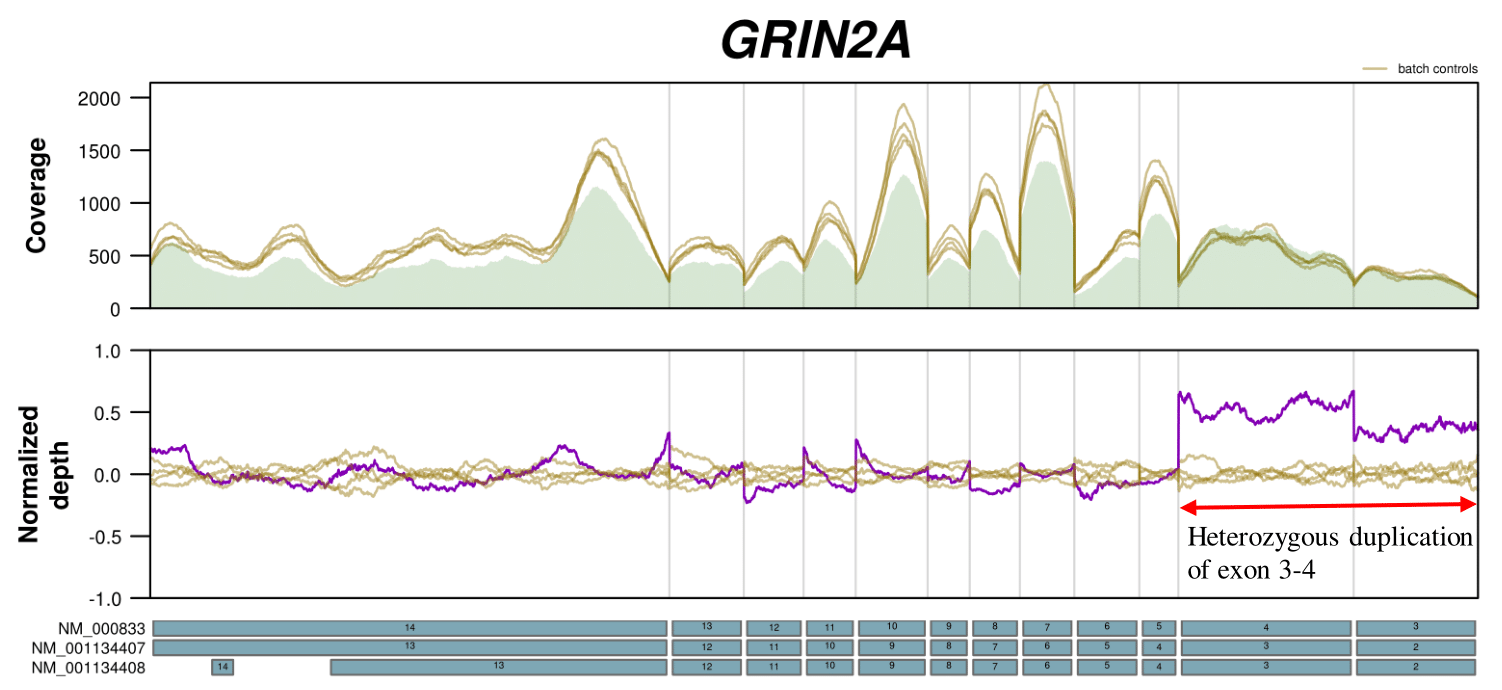

Supplement: Supplementary file 1 [file Data_Sheet_1.docx]
